# Impacts of Mootral on Methane Production, Rumen Fermentation, and Microbial Community in an *in vitro* Study

**DOI:** 10.3389/fvets.2020.623817

**Published:** 2021-01-22

**Authors:** Eslam Ahmed, Rintaro Yano, Miho Fujimori, Deepashree Kand, Masaaki Hanada, Takehiro Nishida, Naoki Fukuma

**Affiliations:** ^1^Graduate School of Animal Husbandry, Obihiro University of Agriculture and Veterinary Medicine, Obihiro, Japan; ^2^Department of Animal Behavior and Management, Faculty of Veterinary Medicine, South Valley University, Qena, Egypt; ^3^Mootral GmbH, Berlin, Germany; ^4^Department of Life and Food Sciences, Obihiro University of Agriculture and Veterinary Medicine, Obihiro, Japan; ^5^Research Center for Global Agromedicine, Obihiro University of Agriculture and Veterinary Medicine, Obihiro, Japan

**Keywords:** mootral, methane emission, rumen, bacteria, archaea

## Abstract

Methane mitigation strategies have a two-sided benefit for both environment and efficient livestock production. This preliminary short-term *in vitro* trial using Mootral (garlic and citrus extracts), a novel natural feed supplement, was conducted to evaluate its efficacy on rumen fermentation characteristics, methane production, and the bacterial and archaeal community. The experiment was performed as a batch culture using rumen fluid collected from sheep, and Mootral was supplemented in three concentrations: 0% (Control), 10%, and 20% of the substrate (50% Grass:50% Concentrate). The rumen fermentation data and alpha diversity of microbial community were analyzed by ordinary one-way analysis of variance. The relative abundance and statistical significance of families and operational taxonomic units (OTUs) among the groups were compared by Kruskal–Wallis H test using Calypso software. After 24-h incubation at 39°C, Mootral in a dose-dependent manner improved the production of total volatile fatty acids and propionate while it reduced the acetate proportion and acetate/propionate ratio. The total produced gas was two times higher in the Mootral-supplemented groups than control (*P* < 0.01), while the proportion of methane in the produced gas was reduced by 22% (*P* < 0.05) and 54% (*P* < 0.01) for 10 and 20% Mootral, respectively. Mootral did not change pH, digestibility, and ammonia-nitrogen. Microbial community analyses showed that Mootral effectively changed the ruminal microbiome. The bacterial community showed an increase of the relative abundance of the propionate-producing family such as *Prevotellaceae* (*P* = 0.014) and *Veillonellaceae* (*P* = 0.030), while there was a decrease in the relative abundance of some hydrogen-producing bacteria by Mootral supplementation. In the archaeal community, *Methanobacteriaceae* was decreased by Mootral supplementation compared with control (*P* = 0.032), while the *Methanomassiliicoccaceae* family increased in a dose-dependent effect (*P* = 0.038). The results of the study showed the efficacy of the new mixture to alter the ruminal microbial community, produce more propionate, and reduce microbial groups associated with methane production, thus suggesting that Mootral is a promising natural mixture for methane reduction from ruminants.

## Introduction

Within livestock, ruminants are blamed to be the main contributors to greenhouse gas production, estimated approximately 80% of the total sector's emissions through enteric fermentation and about 14.5% of total anthropogenic greenhouse gas emissions (1). Enteric methane (CH_4_) is produced by methanogenic archaea found mainly in the rumen, where they convert the hydrogen (H_2_) and carbon dioxide (CO_2_) produced from fermentation by a complex community of ciliate protozoa, bacteria, and anaerobic fungi to CH_4_ (2). On the other hand, microbiome analysis has identified that numerous bacteria seem to be associated with variations in CH_4_ production in ruminants (3). This enteric CH_4_ emission is also associated with a dietary energy loss of 2–12%, hence reduced feed efficiency (4). Therefore, due to the negative environmental and animal production impacts, CH_4_ mitigation has come forward in the last few decades (5).

To date, numerous efforts were made in order to reduce CH_4_ emission from ruminants. These mitigation strategies include rumen manipulation, alteration of rumen fermentation, and modification of rumen microbial biodiversity by different means. Dietary manipulation is directly linked to changes in the rumen fermentation pattern and types of end products. A review highlighted that changing fermentation pattern is one of the most effective ways of CH_4_ abatement (5). Researchers in the field of animal husbandry and nutrition are focusing on usage of natural products such as plant secondary metabolites (PSM) as environmentally safe alternatives to synthetic chemicals in ruminants' ration since the ban on the use of antibiotics and chemicals as a feed supplement in animals' feed (6). Researches have shown the ability of PSM to improve microbial activity and reduce CH_4_ production through decreasing the number of ciliated protozoa and inhibiting methanogenic archaea (7–9).

Bioactive components extracted from garlic (*Allium sativum*), including several sulfur-containing compounds such as alliin, diallyl sulfides, and allicin, have been known for their antimicrobial efficacy (10) and studied to show their potentiality to reduce CH_4_ production through direct inhibition of ruminal archaea (11). However, results showed some variations in CH_4_ reduction capacity ranging from no reduction (12, 13) to 38.5% reduction (14). Besides, flavonoids are one of the important phytochemicals found in most citrus fruits and vegetables that have shown the potentiality to suppress CH_4_ production (15). However, there is still rare information about flavonoid effects on rumen fermentation profile, with growing interest in the usage of flavonoids as natural feed supplements in ruminant feed (16).

Researchers are searching for new mixtures by combining many natural anti-methanogenic compounds that could be able to suppress CH_4_ production effectively without impairing digestibility and volatile fatty acids (VFA) production (17). Accordingly, Mootral (Mootral SA, Rolle, Switzerland), a novel combination of garlic (*A. sativum*) powder and bitter orange (*Citrus aurantium*) extracts, has shown a great efficacy in reduction of CH_4_ from ruminant through alteration of the archaeal community without impairing VFA production when used as a feed supplement in *in vitro* trial with 70% hay:30% concentrate diet using rumen fluid from cows (18).

However, there is still a limitation to find out its ability in different dietary regimen and in another ruminant species, as well as its impact on not only archaeal but also bacterial community in the rumen. Therefore, this preliminary small-scale *in vitro* study was performed using rumen fluid collected from sheep to evaluate the effect of Mootral supplemented to a 50% hay:50% concentrate diet on suppressing CH_4_ production, alteration of ruminal microbiome (Bacterial and Archaeal), as well as rumen fermentation profile and digestibility.

## Materials and Methods

### Rumen Fluid Collection

Rumen fluid was collected from three Corriedale wether sheep (body weight, 63.5 ± 4.4 kg) 4 h after the morning feeding using a stomach tube and vacuum pump. They were fed the basal diet of 50% concentrate and 50% Kleingrass (*Panicum coloratum*) hay at maintenance level for energy requirement. The first amount (100 ml) of the sucked rumen fluid was discarded to prevent contamination of saliva. The second amount was strained by four layers of absorbent gauze into an insulated container and transferred immediately to the laboratory. In the laboratory, the collected samples were mixed together in one beaker under a constant stream of CO_2_ and kept in a water bath at 39°C prior to adding into the fermentation tubes. Animal management and sampling procedures were approved by the Animal Care and Use Committee of the Obihiro University of Agriculture and Veterinary Medicine (Approval Number 19–94).

### Experimental Design and *in vitro* Incubation

The chemical composition of the feed used in the *in vitro* experiment is described in Table 1. The chemical composition of dry matter (930.15), organic matter (942.05), crude protein (984.13), and ether extract (920.39) was determined according to AOAC (19). Neutral detergent fiber, acid detergent fiber, and acid detergent lignin were measured and expressed inclusive of residual ash using an ANKOM^200^ Fiber Analyzer (Ankom Technology Methods 6, 5, and 8, respectively; ANKOM Technology Corp., Macedon, NY, USA). The neutral detergent fiber was measured using sodium sulfite without heat-stable α-amylase. Three groups with three replicates for each were prepared: control (0% Mootral of substrate), 10% (10% Mootral of substrate), and 20% (20% Mootral of substrate). Each group is composed of 900 mg of ground feed (Concentrate + Kleingrass hay) as substrate added in a ratio of 50:50 in a sealed nylon bag (BG1020, Sanshin Industrial Co., Ltd., Kanagawa, Japan) that was placed in a 200-ml fermentation bottle. Feed was ground by a mill to pass through a 1-mm sieve. Mootral was used as a feed supplement added directly in the fermentation bottles in either 10% or 20% of the substrate. Mootral is composed of a mixture of nine parts garlic powder to one part citrus powder. The chemical composition of Mootral is described in Table 2. More information about formulation of Mootral has been published by Eger et al. (18). Thirty milliliters of rumen fluid and 60 ml of artificial saliva (20) were added in the fermentation bottles under continuous flushing with CO_2_. Thereafter, tubes were sealed with rubber stoppers and aluminum caps. The incubation was performed for 24 h at 39°C. At the end of incubation, the produced headspace gas was measured using syringe. Culture fluid was used for measuring pH using pH meter (LAQUA F-72, HORIBA Scientific, Kyoto, Japan), and an aliquot was transferred into 1.5-ml tubes and centrifugated at 16,000 × *g* at 4°C for 5 min. The supernatant and precipitation were collected and stored at −20°C.

**Table 1:** Chemical composition of the feed used in this study (g/kg dry matter).

**(g/kg dry matter)**	**Concentrate**	**Kleingrass hay**
Dry matter (in fresh matter)	837.9	849.6
Organic matter	942.0	916.2
Crude ash	58.0	83.8
Crude protein	229.3	135.8
Ether extract	40.1	38.1
Neutral detergent fiber	281.8	680.0
Acid detergent fiber	98.2	329.9
Acid detergent lignin	25.2	50.0

**Table 2:** Chemical composition of Mootral powder (g/100 g dry matter).

**(g/100 g dry matter)**	**Mootral**
Crude ash	3.9
Crude protein	22.0
Crude fat	0.51
Crude fiber	1.9
Sodium	0.04

### *In vitro* Dry Matter Digestibility

Bags from each tube were washed by running tap water until the drain drops were clear. After that, they were dried in the oven at 60°C for 48 h to determine the *in vitro* dry matter digestibility (IVDMD) (21).

### Gas Composition, VFA, and NH_3_-N Analysis

The headspace gas samples were analyzed by injection of 1 ml of the gas using Hamilton gastight syringe (Hamilton Company, Reno, Nevada, USA) in a gas chromatograph (GC-8A, Shimadzu Corp., Kyoto, Japan) as described previously (22).

VFA were analyzed using high-pressure liquid chromatography (HPLC) (23). Briefly, the analytical specifications were as follows: column, Shim-pak SCR-102H (7 mm, i.d. 8.0 mm × 300 mm, Shimadzu Corp., Kyoto, Japan); eluent flow rate and mobile phase for organic acid analysis (Shimadzu Corp., Kyoto, Japan) at 0.8 ml/min; column temperature, 40°C; reaction reagent and flow rate, pH buffer for organic acid analysis (Shimadzu Corp., Kyoto, Japan) at 0.8 ml/min; conductivity detector (CDD-10AVP, Shimadzu Corp., Kyoto, Japan). Quantification of VFA concentration was performed using the external standard quantitation method.

For measuring the concentration of NH_3_-N, samples were diluted 100 times using 0.1 M phosphate buffer (pH 5.5) and then analyzed according to the Modified Fujii-Okuda method (24) using NH_3_ kit (FUJIFILM Wako Pure Chemical Corp., Osaka, Japan).

### DNA Extraction, Analysis of 16S Ribosomal RNA (16S rRNA), and Next-Generation Sequencing

DNA was extracted from rumen fluid samples using repeated beads beating plus column (RBB+C) method and the Maxwell 16 LEV blood DNA kit (Promega, Madison, WI, USA) (25, 26). The concentration and purity of extracted DNA were measured by a NanoDrop 2000c spectrophotometer (Thermo Fisher Scientific, Tokyo, Japan), and then the DNA concentration was adjusted to 5 ng/μl using Tris-EDTA buffer. The variable regions (V3 and V4) of bacterial 16S rRNA gene were amplified from the purified DNA. The primers used in this study consisted of the Illumina overhang adapters and universal primers in the first stage of PCR as follows: the forward overhang adapter and bacterial universal primer 5′-TCGTCGGCAGCGTCAGATGTGTATAAGAGACAGCCTACGGGNGGCWGCAG-3′ and the reverse overhang adapter and bacterial universal primer 5′-TCTCGTGGGCTCGGAGATGTGTATAAGAGACAGGATTACHVGGGTATCTAATCC-3′. For Archaeal 16S rRNA gene, the forward overhang adapter and Arch349F (5′-TCGTCGGCAGCGTCAGATGTGTATAAGAGACAGGYGCASCAGKCGMGAAW-3′) and the reverse overhang adapter and Arch806R (5′-GTCTCGTGGGCTCGGAGATGTGTATAAGAGACAGGGACTACVSGGGTATCTAAT-3′) were used. In the second PCR, Illumina sequencing adapters and dual index barcodes were added to the amplicons using Nextera® XT Index Kit (Illumina Inc., San Diego, California, USA). The concentration of the second PCR product was quantified using a Quantus™ fluorometer (QuantiFluor® dsDNA System, Promega, Madison, Wisconsin, USA) and then PCR products from all samples were pooled in one tube in equal amounts. Paired-end sequencing was performed using the Illumina MiSeq (Illumina, San Diego, California, USA). The preparation of 16S rRNA gene amplicon was done as previously described by Pelpolage et al. (27).

The analysis of raw 16S rRNA gene sequence was done according to the method described before by Warren et al. (25). Samples with < 1,000 sequence reads were removed. Sequence reads were clustered into operational taxonomic units (OTUs) with a 97% sequence identity threshold. The generated biome table was used in Calypso version 8.84 to generate a principal coordinate analysis (PCoA) 3D plot and express relative abundance of bacterial or archaeal taxa among experimental groups. For the archaeal community, samples were rarefied to a read depth of 1,678, while in the bacterial community, there were 16,246 reads. Nucleotide sequence data reported in this study are available in the DDBJ Sequence Read Archive under the accession numbers DRA011192.

### Statistical Analysis

All rumen fermentation profile data and alpha diversity of both the bacterial and archaeal community were analyzed statistically using GraphPad Prism 8.0.1 (GraphPad Software, San Diego, California, USA). Data were provided as means ± SEM (standard error of the mean). Ordinary one-way analysis of variance (ANOVA) was performed followed by Tukey's test to find the significance among experimental groups. The relative abundance and statistical significance of families and OTU among the three experimental groups were compared using Kruskal–Wallis H test in Calypso version 8.84. Differences were considered statistically significant when *P*-value was < 0.05, and tendency was declared when *P*-value was between 0.05 and 0.10.

## Results

### Effect of Mootral on Rumen Fermentation Characteristics

**Figure 1:**
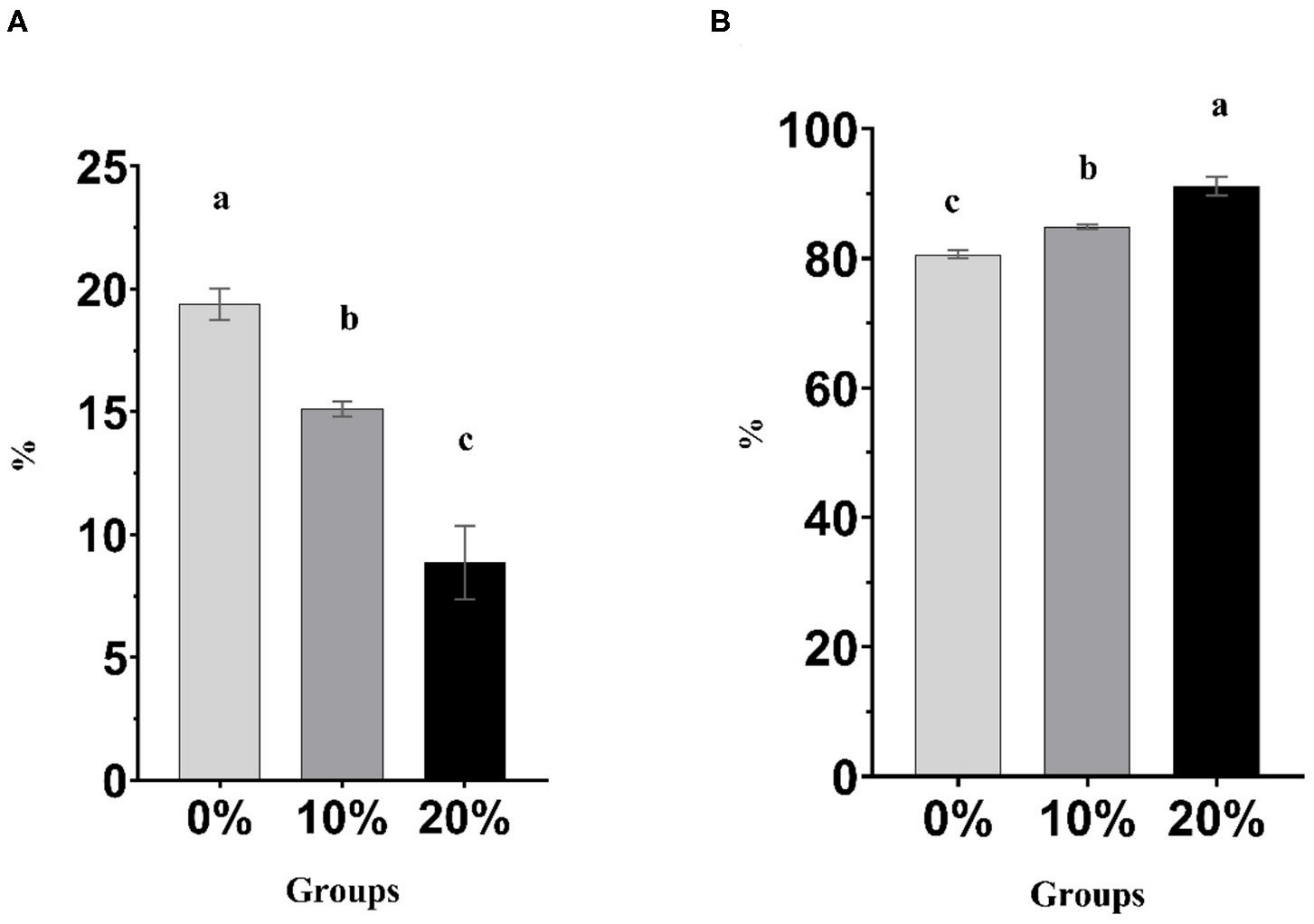
**(A)** Percentage of CH_4_ in the produced gas. **(B)** Percentage of CO_2_ in the produced gas. Bars with different superscripts are different (*P* < 0.05).

Mootral exhibited significant changes in the rumen fermentation profile as shown in Table 3. However, pH, IVDMD, and NH_3_-N did not show any differences (*P* > 0.05) among experimental groups. The total gas produced was increased (*P* < 0.01) for Mootral-supplemented groups compared with control. In contrast, the percentage of the volume of CH_4_ in the produced gas was decreased by increasing Mootral dose; it showed a reduction of up to 21.98% for 10% Mootral (*P* < 0.05) and 54.25% for 20% Mootral (*P* < 0.01) when compared with control (Figure 1A). However, the volume of the produced CH_4_ per day and CH_4_/DM were not different between Mootral-treated groups and control (*P* > 0.05), but there was an increase in 10% compared to 20% (*P* < 0.05) (Table 3). The percentage of the volume of CO_2_ in the produced gas was increased by increasing Mootral supplementation. It was higher for 20% when compared with control (*P* < 0.01) and with 10% (*P* < 0.05), and it was also higher for 10% compared with the control group (*P* < 0.05) (Figure 1B). Consequently, the total amount of CO_2_ produced in 24 h was higher (*P* < 0.01) by increasing Mootral dosages (69.54 and 76.51 ml for 10% and 20%, respectively) when compared with control (35.01 ml) (Table 3). The CH_4_/CO_2_ ratio in the total produced gas decreased with increasing Mootral dosage (*P* < 0.01) (Table 3).

**Table 3:** Rumen fermentation characteristics of 24-h *in vitro* incubation.

**Parameter**	**Experimental groups**			***P*-value**
	**0%**	**10%**	**20%**	
pH	6.87 ± 0.04	6.86 ± 0.01	6.80 ± 0.01	0.13
Total gas production (ml)	43.43 ± 1.29^b^	81.93 ± 2.56^a^	84.03 ± 2.32^a^	<0.01
IVDMD^1^ (%)	62.70 ± 4.70	59.10 ± 1.40	59.00 ± 3.30	0.70
CH_4_ (ml)	8.42 ± 0.41^ab^	12.39 ± 0.52^a^	7.52 ± 1.48^b^	0.02
CH_4_/DM (ml/g)	11.12 ± 0.54^ab^	16.39 ± 0.68^a^	9.89 ± 1.95^b^	0.02
CO_2_ (ml)	35.01 ± 1.01^c^	69.54 ± 2.12^b^	76.51 ± 0.87^a^	<0.01
CH_4_/CO_2_ ratio	0.241 ± 0.01^a^	0.178 ± 0.00^b^	0.098 ± 0.02^c^	<0.01
Acetate (mmol/L)	48.22 ± 1.70	48.13 ± 0.26	48.85 ± 0.33	0.86
Propionate (mmol/L)	21.77 ± 1.21^b^	24.83 ± 0.28^b^	30.28 ± 0.94^a^	<0.01
Butyrate (mmol/L)	11.56 ± 0.86	12.33 ± 0.24	13.03 ± 1.24	0.54
Total VFA^2^ (mmol/L)	81.55 ± 3.64^b^	85.29 ± 0.73^ab^	92.16 ± 0.68^a^	0.04
Acetate (%)	59.18 ± 0.79^a^	56.44 ± 0.27^b^	53.01 ± 0.08^c^	<0.01
Propionate (%)	26.67 ± 0.60^b^	29.11 ± 0.12^b^	32.87 ± 1.24^a^	<0.01
Butyrate (%)	14.14 ± 0.43	14.45 ± 0.17	14.12 ± 1.26	0.95
A/P ratio^3^	2.22 ± 0.08^a^	1.94 ± 0.02^b^	1.62 ± 0.06^c^	<0.01
NH_3_-N (mg/dl)	11.07 ± 1.38	11.51 ± 0.84	10.33 ± 0.20	0.69

a, b, c *Values within the same row with different superscripts are different (P <0.05)*.

1 *IVDMD, in vitro dry matter digestibility*;

2 *VFA, volatile fatty acids*;

3 *A/P, acetate/propionate ratio. ±, standard error of the mean*.

Acetic acid ratio showed a reduction among groups (*P* < 0.01) with increasing Mootral supplementation. However, the propionic acid concentration and ratio increased with increasing Mootral supplementation with a significant effect between 20% and control (*P* < 0.01) and between 20% and 10% (*P* < 0.05). Mootral did not affect either the concentration or percentage of butyric acid among groups. The total VFA increased with Mootral supplementation where it reached a significant level between 20% and the control group (*P* < 0.05). The acetate/propionate (A/P) ratio decreased with increasing Mootral dosage (*P* < 0.01) (Table 3).

### Effect of Mootral on Bacterial Community Diversity and Composition

**Figure 2:**
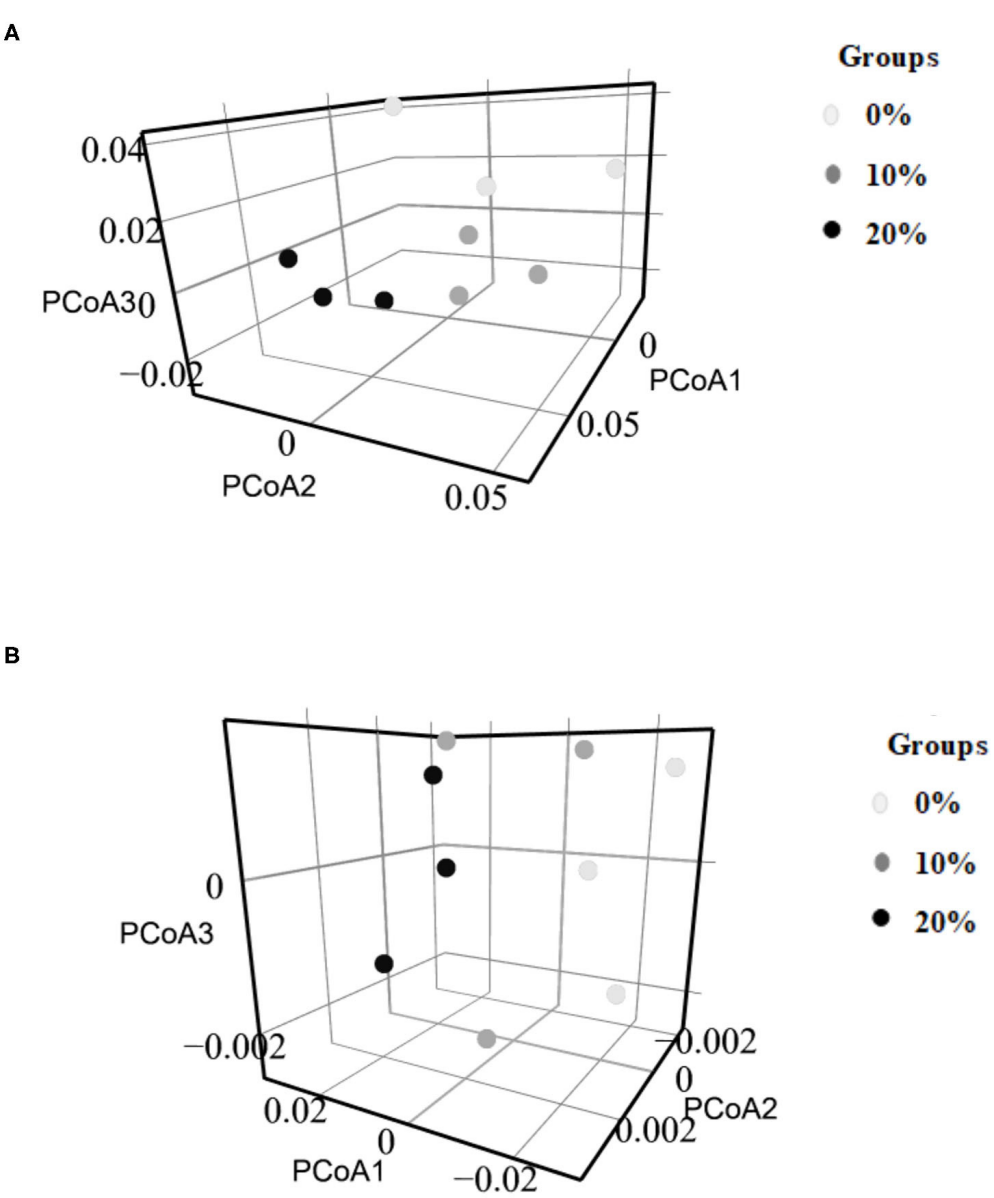
Beta diversity of microbial community analysis by PCoA 3D. **(A)** Bacterial community (Family level), **(B)** Archaeal community (Family level).

The minimum and maximum sequence reads were 16,246 and 42,030, respectively. The α-diversity indices including Richness, Choa1, Evenness, and Shannon index were not affected by Mootral supplementation (*P* > 0.05) (Table 4). However, based on β-diversity analysis at the family level, the control samples were clustered away from Mootral-supplemented groups. Also, Mootral treated samples were clustered close to each other (Figure 2A).

**Table 4:** α-Diversity of microbial community based on OTU level.

**Parameter**	**Experimental groups**			***P*-value**
	**0%**	**10%**	**20%**	
**Bacteria^a^**				
Richness	992.60 ± 7.63	1004.00 ± 5.27	973.60 ± 9.59	0.08
Chao1	1035.00 ± 0.73	1043.00 ± 0.12	1034.00 ± 4.94	0.14
Evenness	0.825 ± 0.01	0.840 ± 0.01	0.823 ± 0.01	0.17
Shannon index	5.72 ± 0.06	5.84 ± 0.04	5.69 ± 0.05	0.14
**Archaea^b^**				
Richness	226.70 ± 20.26	185.90 ± 28.40	232.60 ± 13.37	0.32
Chao1	408.70 ± 7.20	326.70 ± 60.54	406.70 ± 8.60	0.25
Evenness	0.742 ± 0.24	0.731 ± 0.02	0.750 ± 0.01	0.81
Shannon index	4.43 ± 0.17	4.09 ± 0.26	4.45 ± 0.09	0.33

a *α-Diversity of bacteria of the experimental groups. Samples rarefied to a read depth of 16,246*.

b *α-Diversity of archaea of the experimental groups. Samples rarefied to a read depth of 1,678. ±, standard error of the mean*.

**Figure 3:**
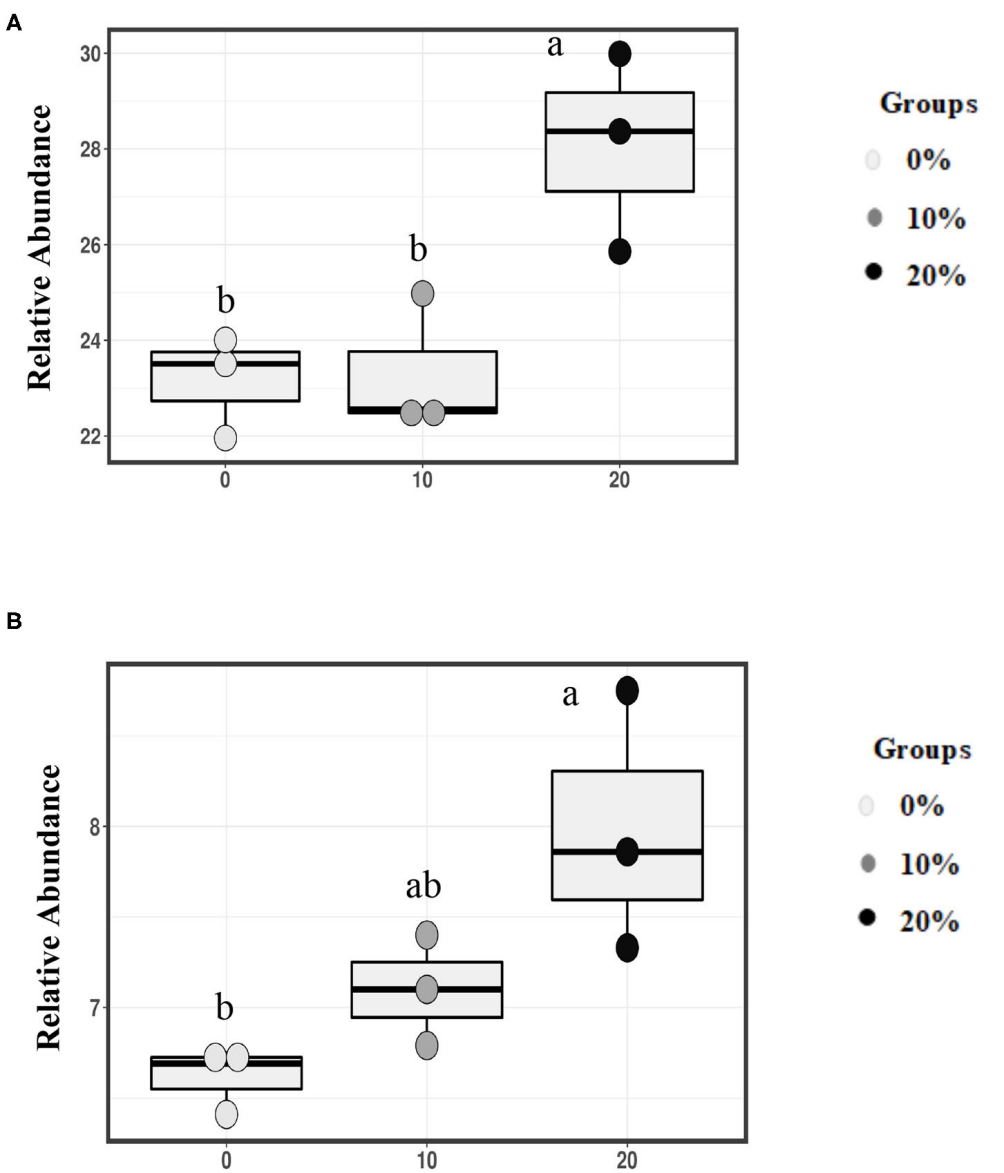
Relative abundance of families. **(A)** *Prevotellaceae*, and **(B)** *Veillonellaceae* in total bacterial community. Boxes with different superscripts are different (*P* < 0.05).

Based on family level, Mootral was able to change the bacterial composition by increasing the relative abundance of family *Prevotellaceae* especially in 20% Mootral (28.07%); this increase was significant when compared with the control group (23.16%) (*P* < 0.05) and with 10% Mootral (23.32%) (*P* < 0.05), while there was no difference between 10% Mootral and control (*P* = 0.99) (Figure 3A). Similarly, the relative abundance of the family *Veillonellaceae* increased (*P* < 0.05) in 20% Mootral (7.98%) compared with control (6.62%), while the abundance of *Veillonellaceae* in 10% Mootral (7.1%) was not statistically different from that in control (*P* = 0.46) (Figure 3B). Based on OTU level, some bacterial strain belonging to family *Ruminococcaceae* and order *Bacteroidales* significantly decreased between Mootral-supplemented groups and control (*P* < 0.05) (Supplementary Figure 1). The relative abundance of all taxa at the phylum and family level of bacteria, which was more than 0.1% of total bacteria, was described in Supplementary Table 1.

### Effect of Mootral on Archaeal Community Diversity and Composition

The minimum sample sequence read was 1678 and the maximum was 19,986. Mootral supplementation did not alter the α-diversity indices of the archaeal community among experimental groups (Table 4). However, the β-diversity analysis at the family level showed a shift of the control group samples to be away from Mootral-supplemented samples (Figure 2B).

**Figure 4:**
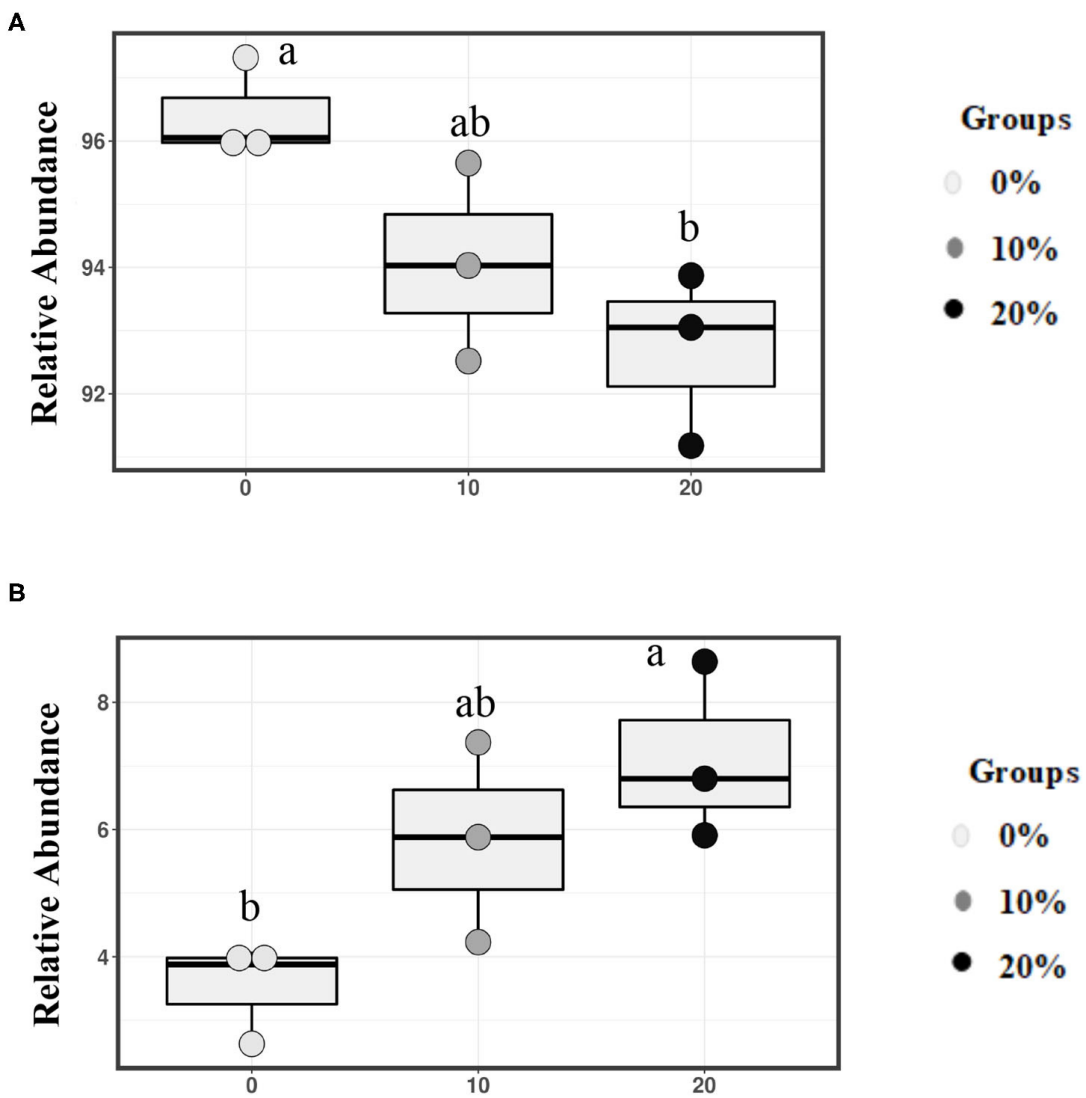
Relative abundance of families. **(A)** *Methanobacteriaceae*, and **(B)** *Methanomasillicocae* in total archaeal community. Boxes with different superscripts are different (*P* < 0.05).

Mootral-supplemented groups showed a significant shift in the archaeal community by decreasing the relative abundance of the major methanogenic group, family *Methanobacteriaceae* (94.07 and 92.70% for 10 and 20% Mootral, respectively), when compared with the control group (96.42%) (*P* < 0.05) (Figure 4A). In contrast, the relative abundance of family *Methanomassiliicoccaceae* increased with increasing Mootral dosages (5.83 and 7.12% for 10 and 20% Mootral, respectively) when compared with non-supplemented group (3.5%) (*P* < 0.05) (Figure 4B). The relative abundance of all taxa at the phylum and family level of archaea that was more than 0.1% of total archaea was described in Supplementary Table 1.

## Discussion

Previous studies have shown the efficacy of garlic compounds and flavonoids on CH_4_ suppressing of either *in vivo* or *in vitro* studies (15, 28, 29); however, there are still variations on VFA productions and digestibility (15, 30–32). Combination of these two natural products may have a significant effect on reducing CH_4_ without impairing rumen fermentation characteristics. To our knowledge, only two studies were performed using that combination of garlic and citrus (Mootral) to evaluate its effect on CH_4_ emission (33) and fermentation profile through alterations of the archaeal community (18), while the bacterial community has not been investigated yet. Further investigations are required to ensure efficacy in different diet forms and different ruminant species. Therefore, this preliminary *in vitro* trial was performed to evaluate that efficacy in a different feeding regimen (50% Kleingrass hay:50% Concentrate) and another ruminant species (sheep) through studying its impacts on the rumen fermentation profile and archaeal and bacterial communities.

### Mootral Improved Rumen Fermentation Characteristics by Reducing the Percentage of Methane

Most of the anti-methanogenic products studied before showed negative effects on fermentation profile at high doses to achieve the effective CH_4_ reduction (11, 17, 34). However, the results of the current study showed an increase in propionic acid and total VFA with increasing Mootral dosages that may be due to stimulation of the family *Prevotellaceae* that increased in the current study. *Prevotellaceae* is well-known as a propionate-producing bacteria (35). Many researches have proved that fermentation leading to more propionate is strongly associated with decrease in CH_4_ production. For instance, Ungerfeld (36) reported that reduction of CH_4_ in batch cultures leads to redirection of metabolic H_2_ toward propionate production_._ Similarly, Kittelmann et al. (37) assumed that high propionate was present in low-CH_4_-emitting cows. The improvement in the production of total VFA by Mootral effect was also observed in a previous study (18) and in other studies that used either garlic (38) or flavonoids (39). The reduction of acetate and A/P ratios was also reported with the inclusion of 300 mg/L garlic oil in a continuous culture system (40) and garlic powder (30). Production of more propionate and less acetate means that H_2_ was redirected toward propionate formation as an alternative way other than methanogenesis (41). The rumen medium was stabilized in the presence of garlic and citrus and did not change the pH, IVDMD, and NH_3_-N as shown previously (39, 42, 43) and similarly when the same mixture was used (18).

Mootral supplementation showed a strong efficacy to reduce the CH_4_ percentage in the produced gas up to 54%. Interestingly, the total gas produced (ml/day) was two-fold more in Mootral-supplemented groups than control and that explains why the total CH_4_ production/day was not changed between Mootral groups and the control. The trial data of an *in vitro* gas production monitoring for 48 h from Copenhagen University (44) showed a similar finding to our results. Mootral stimulated fermentation, and the total gas production increased during the first 16 h. Also, they reported that Mootral reduced the percentage of CH_4_ in the produced gas with 58% from a typical Danish dairy feed ration without affecting IVDMD. The substantial increase in total gas production and CO_2_ with reducing CH_4_/CO_2_ ratio as an important indicator of rumen fermentation profile in the current study might be due to the stimulating effect of Mootral on the activity of some rumen microbes other than methanogens. This interesting finding has to be proven in further researches using qPCR. Eger et al. (18) reported that Mootral reduced the percentage and the total production of CH_4_ in the RUSITEC system as a long-term study (14 and 18 days). As our present study was a batch culture for short term (24 h), these differences could contribute to the result's discrepancy.

### Mootral Changed the Bacterial Community Composition

Although Mootral was effective in shifting the bacterial community toward less H_2_-producing bacteria, the α-diversity was not different. By analyzing the bacterial community of ruminants, it has been proven that differences in the bacterial community composition were associated with the level of CH_4_ emissions (37). The results of the current study revealed that *Prevotellaceae*, the main dominant family in rumen fluid (45), was higher with Mootral groups especially the higher dose (20%) as mentioned previously. The genus *Prevotella* is well-known to produce propionate by utilizing H_2_ *via* the randomizing (succinate) or non-randomizing (acrylate) pathways through the fermentation of carbohydrates. These pathways were the main ways for consumption of H_2_, which accumulated as a consequence of reduced methanogenesis (35). Similarly, the family *Veillonellaceae* showed a higher abundance in Mootral groups. This might be due to the effect of flavonoids extracted from citrus (15). *Megasphaera elsdenii* belonging to the family *Veillonellaceae* is well-known as lactate-utilizing and propionate-producing bacteria (46). Moreover, *Quinella* spp., a member of the family *Veillonellaceae*, were more numerous in low-CH_4_-producing cows (47). Tapio et al. (3) also reported that the lower CH_4_ production ruminotype possessed a high relative number of propionate-producing *Quinella ovalis* and succinate-producing bacteria such as *Prevotella bryantii*.

Within bacteria, some species belonging to *Ruminococcaceae, Clostridiales*, and *Bacteroidales* are H_2_ producers, while *Prevotella* spp. are net H_2_ utilizers (48). Denman et al. (35) attributed that CH_4_ emission depends on the abundance of H_2_-producing and -consuming bacteria. The results from the current study based on OTU level showed a lower abundance of some OTUs belonging to *Ruminococcaceae and Bacteroidales*, while some OTUs belonging to genus *Prevotella* were higher in Mootral-treated groups compared to the control group. Similar findings were observed by Popova et al. (49) who found that reduction of CH_4_ by linseed and nitrate reduced the relative abundance of *Ruminococcaceae* as well, and linseed supplementation increased the proportion of *Prevotellaceae*. The combination effect of Mootral on the bacterial community is still unclear, but it could have an indirect effect on increasing the relative abundance of H_2_-utilizing bacteria by reducing methanogenesis and stimulating the utilization of accumulated H_2_ by those bacteria to produce propionate. There is a need to further understand the mode of action of Mootral to these bacteria in upcoming researches.

### Mootral Altered the Archaeal Community Composition

Similar to the results in the bacterial community, the archaeal α-diversity did not show any changes with Mootral supplementation, which was similar to previous findings (18). The archaeal sequences were assigned to two dominant families, *Methanobacteriaceae* and *Methanomassiliicoccaceae*. These two families were also dominant in other 16S rRNA gene-based studies (50–52). Mootral showed significant changes in the archaeal community by decreasing the relative abundance of the dominant family group *Methanobacteriaceae*. The family *Methanobacteriaceae* includes the genus *Methanobrevibacter*, which is a well-known major CH_4_ producer in the rumen (53, 54). The reduction of the family *Methanobacteriaceae* might be related to the direct effect of organosulfur compounds of garlic in Mootral through interaction with cell membrane and inhibiting certain SH-containing enzymes essential for metabolic activities of methanogenic archaea (11). The effect of garlic on reduction of archaea has also been shown in previous studies (11, 55). Furthermore, it has been reported that flavonoids may have an effect on methanogenic archaea populations (43). Additionally, Oskoueian et al. (15) reported that flavonoids such as naringin and quercetin at the concentration of 4.5% of the substrate suppressed CH_4_ production through reduction of total methanogens. However, researchers are still not aware of the mode of action of flavonoids on archaea. Ruminal ciliated protozoa could enhance the methanogenesis as they are a major H_2_ producer in the rumen, and they act as a host for methanogens. The produced H_2_ is utilized by archaea found either inside or on the external surface of the protozoal cells (56). *Methanobacteriaceae* with its species were found to be associated with protozoa (57, 58). It has been shown that flavonoid supplementation reduced the total protozoal number (15), which has not been investigated in the current study. Thus, further researches are required to study the effect of Mootral on protozoa.

Interestingly, the family *Methanomassiliicoccaceae* showed a higher abundance in Mootral groups in a dose-dependent pattern. St-Pierre and Wright (59) reported that its normal abundance within archaea in the rumen is about 5%, which was similar to the results of the current study. To date, as a new archaeal group, information on this taxonomy remains limited (60). A comprehensive understanding of the community of the family could help to know its function in the rumen. However, Danielsson et al. (61) also reported that *Methanomassiliicoccaceae* was 1.5-fold more abundant in low CH_4_ emitters than in high CH_4_ emitters. Moreover, its abundance was higher in a microbial community with low CH_4_ production such as cows supplemented with nitrate (62) as well as in a previous *in vitro* Mootral study (18).

## Conclusion

Mootral supplementation reduced the CH_4_ percentage and CH_4_/CO_2_ ratio in a dose-dependent manner. Mootral was able to shift the fermentation to produce more propionate and less acetate and to increase the production of total VFA without affecting IVDMD. Furthermore, 20% Mootral was effective in increasing the abundance of H_2_-consuming groups such as *Prevotellaceae* and *Veillonellaceae* and in reducing some H_2_-producing bacteria. In addition, the archaeal community was altered by reducing the major CH_4_-producing family *Methanobacteriaceae* and increasing *Methanomassiliicoccaceae*. The results of this study suggest that Mootral as a new combination could have the potentiality to be used for reduction of CH_4_ in ruminants.

## Data Availability Statement

Nucleotide sequence data reported in this study are available in the DDBJ Sequence Read Archive under the accession number DRA011192.

## Ethics Statement

The animal study was reviewed and approved by The Animal Care and Use Committee of Obihiro University of Agriculture and Veterinary Medicine.

## Author Contributions

EA, DK, TN, and NF: conceptualization. EA, DK, and NF: methodology. MH and TN: validation. EA, RY, and MF: formal analysis and investigation. NF and DK: resources. EA: writing—original draft preparation. RY, MF, DK, MH, TN, and NF: writing—review and editing. EA: visualization. MH, TN, and NF: supervision. NF: project administration. All authors have read and agreed to the published version of the manuscript.

## Conflict of Interest

DK was employed by company Mootral GmbH. The remaining authors declare that the research was conducted in the absence of any commercial or financial relationships that could be construed as a potential conflict of interest.
