# Supplementary material for: Impacts of Mootral on Methane Production, Rumen Fermentation, and Microbial Community in an in vitro Study
Source: Front Vet Sci. 2021 Jan 22;7:623817. doi: 10.3389/fvets.2020.623817 (PMC7863759; doi:10.3389/fvets.2020.623817)
Supplement: Supplementary file 1 [file Data_Sheet_1.docx]

**Impacts of Mootral on Methane**

**Production, Rumen Fermentation,**

**and Microbial Community in an**

***in vitro* Study**

Eslam Ahmed^1, 2^, Rintaro Yano^1^, Miho Fujimori^1^, Deepashree Kand^3^, Masaaki Hanada^4^, Takehiro Nishida^4^, Naoki Fukuma^4, 5, *^

^1^ Graduate School of Animal Husbandry, Obihiro University of Agriculture and Veterinary Medicine, Inada, Obihiro 080-8555, Japan.

^2^ Department of Animal Behavior and Management, Faculty of Veterinary Medicine, South Valley University, Qena 83523, Egypt.

^3^ Mootral GmbH, Waldseeweg 6, 13467 Berlin, Germany.

^4^ Department of Life and Food Sciences, Obihiro University of Agriculture and Veterinary Medicine, Inada, Obihiro 080-8555, Japan.

^5^ Research Center for Global Agromedicine, Obihiro University of Agriculture and Veterinary Medicine, Inada, Obihiro 080-8555, Japan.

*Corresponding author: Naoki Fukuma

E-mail: [n.fukumax@obihiro.ac.jp](mailto:n.fukumax@obihiro.ac.jp); Tel : +81-155-49-5463


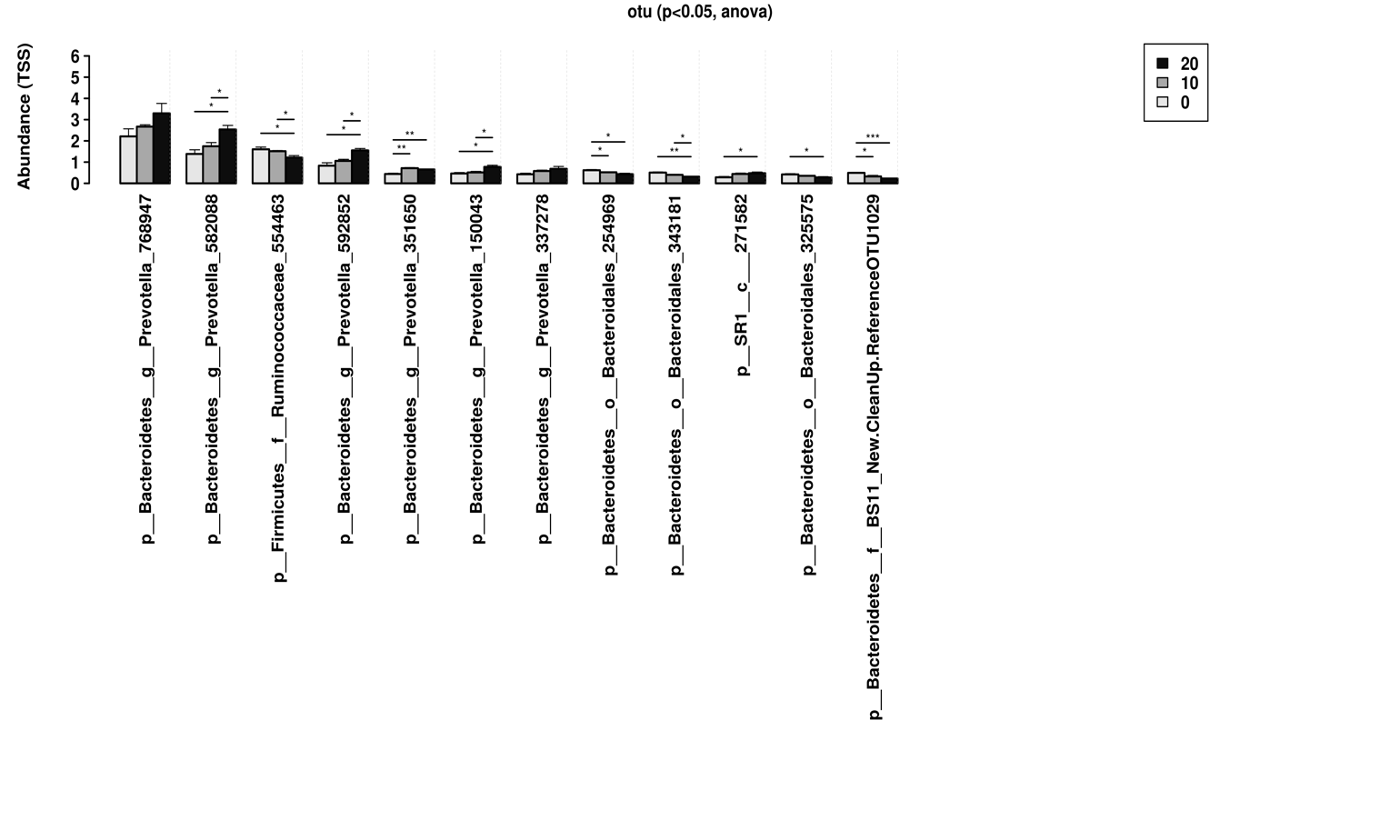
**Supplementary Figure S1:** Relative abundance of bacterial community OTU level, (top 20). Asterisks indicate differences (*P* < 0.05).

**Relative Abundance**

**Groups**

**0%**

**10%**

**20%**

**Table S1:** The mean relative abundance of bacteria and archaea based on phylum and family.

| **Taxa** | **Experimental groups** | | | ***P* value** |
| --- | --- | --- | --- | --- |
|  | **0%** | **10%** | **20%** |  |
| **Bacteria** | | | | |
| **Firmicutes** | 48.33**±**1.35 | 49.62**±**1.65 | 47.38**±**0.38 | 0.49 |
| *Unclassified.Clostridiales* | 18.17**±** 0.76 | 17.15**±** 0.76 | 18.35**±**1.31 | 0.66 |
| *Ruminococcaceae* | 12.00**±**0.32 | 14.05**±**0.53 | 11.12**±**1.12 | 0.08 |
| *Lachnospiraceae* | 6.57**±**0.7 | 6.16**±**0.20 | 6.30**±**0.01 | 0.41 |
| *Veillonellaceae* | 6.62**±**0.11**^b^** | 7.10**±**0.18**^ab^** | 7.10**±**0.41**^a^** | 0.03 |
| *Christensenellaceae* | 0.730**±**0.14 | 0.750**±**0.15 | 0.358**±**0.02 | 0.10 |
| *Clostridiaceae* | 0.757**±**0.06 | 0.837**±**0.07 | 0.730**±**0.09 | 0.59 |
| *Mogibacteriaceae* | 0.817**±**0.03**^a^** | 0.787**±**0.04**^a^** | 0.613**±**0.02**^b^** | 0.01 |
| *Erysipelotrichaceae* | 0.603**±**0.06**^b^** | 0.790**±**0.03**^a^** | 0.747**±**0.03**^ab^** | 0.04 |
| *Streptococcaceae* | 1.95**±**1.09 | 1.88**±**0.39 | 1.14**±**0.11 | 0.66 |
| *Dehalobacteriaceae* | 0.090**±**0.02 | 0.087**±**0.03 | 0.017**±**0.01 | 0.10 |
| **Bacteroidetes** | 42.49**±**0.95 | 43.34**±**1.30 | 46.29**±**0.75 | 0.09 |
| *Prevotellaceae* | 23.16**±**0.62**^b^** | 23.32**±**0.83**^b^** | 28.07**±**1.20**^a^** | 0.01 |
| *Unclassified.Bacteroidales* | 15.40**±**0.74 | 15.11**±**0.45 | 14.65**±**0.25 | 0.61 |
| *Paraprevotellaceae* | 1.50**±**0.18 | 1.58**±**0.03 | 1.36**±**0.11 | 0.50 |
| *BS11* | 1.00**±**0.04 | 1.20**±**0.14 | 0.903**±**0.19 | 0.38 |
| *S247* | 0.457**±**0.01 | 0.580**±**0.11 | 0.393**±**0.02 | 0.19 |

| *RF16* | 0.587**±**0.14 | 1.20**±**0.06 | 0.643**±**0.23 | 0.07 |
| --- | --- | --- | --- | --- |
| *Bacteroidaceae* | 0.370**±**0.01**^a^** | 0.327**±**0.02**^ab^** | 0.253**±**0.02**^b^** | 0.01 |
| **Proteobacteria** | 3.68**±**1.28 | 1.49**±**0.27 | 1.35**±**0.52 | 0.15 |
| *Succinivibrionaceae* | 3.44**±**1.32 | 1.19**±**0.25 | 1.03**±**0.51 | 0.15 |
| *Desulfovibrionaceae* | 0.217**±**0.05 | 0.247**±**0.02 | 0.240**±**0.01 | 0.79 |
| **Spirochaetes** | 0.760**±**0.14 | 0.710**±**0.07 | 0.657**±**0.14 | 0.83 |
| *Spirochaetaceae* | 0.630**±**0.15 | 0.567**±**0.08 | 0.563**±**0.12 | 0.90 |
| **TM7** | 0.757**±**0.02**^ab^** | 0.823**±**0.03**^a^** | 0.703**±**0.02**^b^** | 0.03 |
| *F16* | 0.757**±**0.02**^ab^** | 0.823**±**0.03**^a^** | 0.703**±**0.02**^b^** | 0.03 |
| **SR1** | 0.627**±**0.05**^b^** | 0.887**±**0.07**^ab^** | 0.957**±**0.08**^a^** | 0.03 |
| *Unclassified.SR1* | 0.627**±**0.05**^b^** | 0.887**±**0.07**^ab^** | 0.957**±**0.09**^a^** | 0.03 |
| **Synergistetes** | 0.863**±**0.15 | 0.827**±**0.00 | 0.807**±**0.04 | 0.90 |
| *Dethiosulfovibrionaceae* | 0.863**±**0.15 | 0.827**±**0.00 | 0.807**±**0.04 | 0.90 |
| **Actinobacteria** | 0.783**±**0.15 | 0.717**±**0.07 | 0.737**±**0.04 | 0.88 |
| *Coriobacteriaceae* | 0.783**±**0.15 | 0.717**±**0.07 | 0.737**±**0.04 | 0.88 |
| **Verrucomicrobia** | 0.553**±**0.10 | 0.530**±**0.04 | 0.347**±**0.07 | 0.18 |
| *RFP12* | 0.347**±**0.10 | 0.333**±**0.02 | 0.243**±**0.06 | 0.53 |
| **Chloroflexi** | 0.263**±**0.02 | 0.243**±**0.01 | 0.243**±**0.01 | 0.53 |
| *Anaerolinaceae* | 0.263**±**0.02 | 0.243**±**0.01 | 0.243**±**0.01 | 0.53 |
| **Planctomycetes** | 0.280**±**0.03**^a^** | 0.163**±**0.00**^b^** | 0.147**±**0.02**^b^** | 0.01 |
| *Pirellulaceae* | 0.280**±**0.03**^a^** | 0.163**±**0.00**^b^** | 0.147**±**0.02**^b^** | 0.01 |
| **Tenericutes** | 0.150**±**0.04 | 0.137**±**0.01 | 0.097**±**0.03 | 0.38 |
| *Anaeroplasmataceae* | 0.100**±**0.04 | 0.080**±**0.01 | 0.050**±**0.01 | 0.35 |

| **WPS2** | 0.137**±**0.01**^a^** | 0.1067**±**0.02**^ab^** | 0.063**±**0.00**^b^** | 0.01 |
| --- | --- | --- | --- | --- |
| *Unclassified.WPS2* | 0.137**±**0.013**^a^** | 0.107**±**0.02**^ab^** | 0.063**±**0.00**^b^** | 0.01 |
| **Lentisphaerae** | 0.153**±**0.06 | 0.207**±**0.04 | 0.100**±**0.02 | 0.30 |
| *Victivallaceae* | 0.153**±**0.06 | 0.207**±**0.04 | 0.100**±**0.02 | 0.30 |
| **Archaea** | | | | |
| **Euryarchaeota** | 100 | 100 | 100 | --- |
| *Methanobacteriaceae* | 96.42**±**0.45**^a^** | 94.07**±**0.90**^ab^** | 92.70**±**0.80**^b^** | 0.03 |
| *Methanomassiliicoccaceae* | 3.53**±**0.45**^b^** | 5.83**±**0.91**^ab^** | 7.12**±**0.80**^a^** | 0.04 |
| *Unclassified* | 0.030**±**0.01**^b^** | 0.093**±**0.02**^ab^** | 0.163**±**0.03**^a^** | 0.01 |

**^a,b^** Values within the same row with different superscripts are different (*P* < 0.05). ±: Standard Error of the Mean.
